# Stereo Vision Tracking of Multiple Objects in Complex Indoor Environments

**DOI:** 10.3390/s101008865

**Published:** 2010-09-28

**Authors:** Marta Marrón-Romera, Juan C. García, Miguel A. Sotelo, Daniel Pizarro, Manuel Mazo, José M. Cañas, Cristina Losada, Álvaro Marcos

**Affiliations:** 1 Electronics Department, University of Alcalá, Campus Universitario s/n, 28805, Alcalá de Henares, Madrid, Spain; E-Mails: jcarlos@depeca.uah.es (J.G.); sotelo@depeca.uah.es (M.S.); pizarro@depeca.uah.es (D.P.); mazo@depeca.uah.es (M.M.); losada@depeca.uah.es (C.L.); alvaro.marcos@depeca.uah.es (A.M.); 2 Departamento de Sistemas Telemáticos y Computación, Universidad Rey Juan Carlos, C/Tulipán s/n, 28933, Móstoles, Madrid, Spain; E-Mail: jmplaza@gsyc.es (J.C.)

**Keywords:** 3D tracking, Bayesian estimation, stereo vision sensor, mobile robots

## Abstract

This paper presents a novel system capable of solving the problem of tracking multiple targets in a crowded, complex and dynamic indoor environment, like those typical of mobile robot applications. The proposed solution is based on a stereo vision set in the acquisition step and a probabilistic algorithm in the obstacles position estimation process. The system obtains 3D position and speed information related to each object in the robot’s environment; then it achieves a classification between building elements (ceiling, walls, columns and so on) and the rest of items in robot surroundings. All objects in robot surroundings, both dynamic and static, are considered to be obstacles but the structure of the environment itself. A combination of a Bayesian algorithm and a deterministic clustering process is used in order to obtain a multimodal representation of speed and position of detected obstacles. Performance of the final system has been tested against state of the art proposals; test results validate the authors’ proposal. The designed algorithms and procedures provide a solution to those applications where similar multimodal data structures are found.

## 1. Introduction

Visual tracking is one of the areas of greater interest in robotics as it is related with topics such as visual surveillance or mobile robots navigation. Multiple approaches to this problem have been developed by research community during last decades [1]. Among them, a sorting can be done according to methods used to detect or extract information from the image about objects in the scene:

- With static cameras: background subtraction is generally applied to extract image information corresponding to dynamic objects in the scene. This method is wide spread among the research community [2–4], mainly in surveillance applications.
- With a known model of the object to be tracked: this situation is very common in tracking applications, either using static cameras [3,4] or dynamic ones [5,6]. The detection process is computational more expensive, but the number of false alarms and the robustness of the detector are bigger than if looking for any kind of objects.

**Figure 1.**
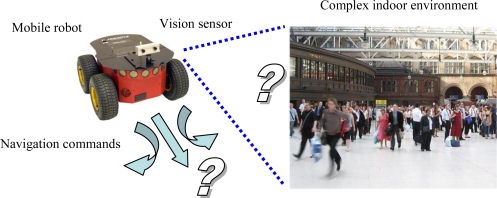
Framework and typical scenario: mobile robot navigation through complex and crowded indoor environments.

All the referred works solve the detection problem quite easily, thanks to the application of the mentioned restrictions. However, an appropriate solution is more difficult to find when the problem to be solved is the navigation of a mobile robot in complex and crowded indoor environments (Figure 1), like museums, railway stations, airports, commercial centers, *etc.* In those scenarios there is any number of dynamic obstacles around and the robot has to detect and track all of them in order to find a suitable path.

In this kind of scenario, both of the standard methods have important drawbacks. When models are used to detect the obstacles, there are problems with the execution time (obstacles can be far away before being identified) and with the modeling of any of the possible objects that could be found in the environment. By the other way, it is not possible to use background subtraction because its visual appearance changes continuously; this is because any element in the visual environment of the robot may be an obstacle, apart from objects that belong to building structures in which the robot is located.

Because the complexity of the information available from a visual sensor, it is convenient to organize first the visual data in the images at least into two classes: measurements coming from obstacles (obstacles class); and measurements coming from the environment (structural features class).

Once this information is available, data classified in the environment class can be used to make a reconstruction of robot surroundings structure. This process is especially interesting for robot navigation, as it can be used in a SLAM (Simultaneous Localization and Mapping [7]) task.

At the same time, data assigned to the obstacles class can be used as an input for any of the tracking algorithms proposed by the scientific community. Taking into account the measurements characteristics, the position tracker has to consider the noise related to them in order to achieve reliable tracking results. Probabilistic algorithms, such as particle filters (PFs, [8–10]) and Kalman filters (KFs, [11,12]), can be used to develop this task as they include this noisy behavior in the estimation process by means of a probabilistic model.

Anyway, the objective is to calculate the posterior probability (also called belief, *p*(*x⃗_t_*|*y⃗*_1:*t*_)) of the state vector *x⃗_t_* and upon the output one *y⃗_t_*, which informs about the position of the target, by means of the Bayes rule, and through a recursive two steps estimation process (prediction-correction), in which some of the involved variables are stochastic.

Most solutions to this multi-tracking problem use one estimator for each object to be tracked [12,13]. These techniques are included in what is called MHT (Multi-Hypothesis Tracking) algorithm. It is also possible to use a single estimator for all the targets if the state vector size is dynamically adapted to include the state variables of the objects’ model as they appear or disappear in the scene [14,15]. Nevertheless, both options are computationally very expensive in order to use them in real time applications.

Then, the most suitable solution is to exploit the multimodality of the probabilistic algorithms in order to include all needed estimations in a single density function. With this idea, a PF is used as a multimodal estimator [16,17]. This idea has not been exploited by the scientific community adducing to the inefficiency of the estimation, due to the impoverishment problem that the PF suffers when working with multimodal densities [18,19].

Anyway, an association algorithm is needed. The association problem is easier if a single measurement for each target is available at each sample time [20]. In contrast, the biggest the amount of information from each model is, the most reliable the estimation will be.

In the work presented here, the source of information is a vision system in order to obtain as more position information from each tracked object as possible. Thus, the needed association algorithm has also a high computational load but the reliability of the tracking process is increased.

The scientific community has tested different alternatives for the association task, including Maximum Likelihood (ML), Nearest Neighbor (NN) and Probabilistic Data Association (PDA) [20]. In our case, we have selected the NN solution due to its deterministic character. Finally, not all proposals referred to in this introduction are appropriate if the number of objects to track is variable: it is necessary an extension of the previously mentioned algorithms.

**Figure 2.**
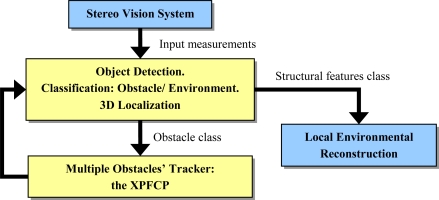
General description of the global stereo vision based tracking system.

In our work, the multimodal ability of the PF is used, and its impoverishment problem is mitigated by using a deterministic NN clustering process that, used as association process, is combined with the probabilistic approach in order to obtain efficient multi-tracking results. We use an extended version of a Bootstrap particle filter [9], called XPFCP (eXtended Particle Filter with Clustering Process), to achieve the position estimation task with a single filter, in real time, and for tracking a variable number of objects detected with the on-board stereo vision process. Figure 2 shows a functional description of the whole tracking application.

Data classified as belonging to the structural features class can be used by standard SLAM algorithms for environmental reconstruction tasks; however, this question is out of the scope of present paper as well as a detailed description of the stereo vision system.

This paper will describe the functionality of the two main processes of the multi-tracking proposal: Section 2 will detail the object detector, classifier and 3D locator; Section 3 will describe the multiple obstacles tracker, the XPFCP algorithm. Section 4 will show the obtained results under a set of testing scenarios. Finally, the paper ends with conclusions about the whole system behavior and the obtained results.

## 2. Detection, Classification and Localization Processes

A stereo vision subsystem is considered as one of the most adequate ways to acquire important information about the different elements found in a dynamic environment. That is because:

- The amount of information that can be extracted from an image is much bigger than the one that can be obtained from any other kind of sensor, such as laser or sonar [21].
- As the environmental configuration changes with time, with a single camera is not possible to obtain the depth coordinate of the objects’ position vector, and thus a stereo vision arrangement is needed.

An alternative to this visual sensor configuration could be to use a Time-Of-Flight (TOF) camera that provides depth information. However, currently these cameras are not available at an affordable price and the information obtained with this sensor is still far from versatile (not valid for long distances) and accurate (post-acquisition process is normally needed in order to compensate reflection effects).

**Figure 3.**
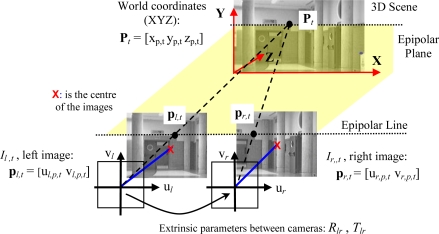
Functional description of the stereo vision data extraction process.

A matching process based on the stereo vision system epipolar geometry allows obtaining the desired 3D position input information [*x_p,t_ y_p,t_ z_p,t_*]*^T^* of a point **P***_t_* from its projections, **p***_l,t_* and **p***_r,t_*, in a pair of synchronized images (*I_l,t_* = [*u_l,p,t_ v_l,p,t_*]*^T^*, *I_r,t_* = [*u_r,p,t_ v_r,p,t_*]*^T^*), as shown in Figure 3.

In this work, the left-right image matching process is solved with a Zero Mean Normalized Cross Correlation (ZNCC), due to its robustness [22]. Each sampling time, *t*, for every pixel of interest (*i.e.*, in the left image), *I_l,t_* = [*u_l,p,t_ v_l,p,t_*]*^T^*), this process consists on looking for a similar gray level among the pixels in the epipolar line at the paired image (the right one *I_r,t_*). 3D location of paired pixels can be found if, after a careful calibration process of both cameras location, the geometric extrinsic parameters of rotation, *R_lr_*, and translation, *T_lr_*, are known.

**Figure 4.**
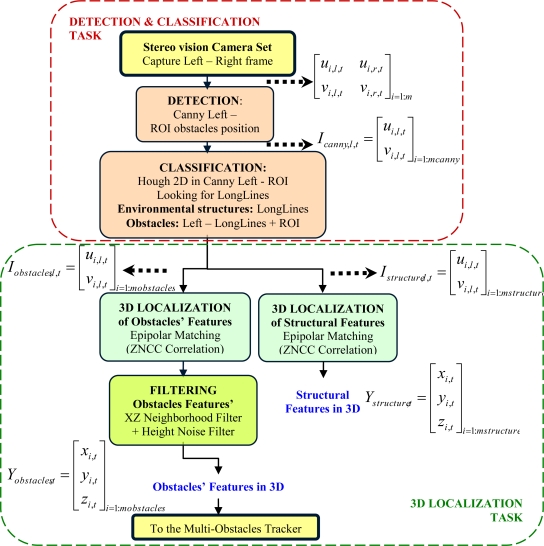
Flowchart of the data acquisition subsystem, based on a stereo vision process. Main tasks are: detection and classification (blocks at the top); and 3D localization (blocks at the bottom). Inner structure of each main task is highlighted and detailed.

As it can be expected, this process is very time consuming. Therefore the 3D information to be obtained should be limited to set of points of interest in both images. In the case of this work, points coming from objects edges have enough information to develop the tracking task. Moreover, just the edges information will enable the possibility of partially reconstructing the structure of the environment in which this tracking is carried out. The global data acquisition process proposed in this paper includes the following main tasks: detection and classification; and 3D localization. Details of these two tasks are shown in Figure 4.

### 2.1. Detection and Classification

The detection and classification process (top group in Figure 4) is executed with each pair of frames (*I_l,t_* and *I_r,t_*) synchronously acquired in sampling time, *t*, from the stereo-camera set. This process is developed through the following steps.

#### 2.1.1. Detection

Edges information is extracted from the pair of cameras with a Canny filter [23]. This information is enough both to track all the objects in the wandering robot environment and partially reconstruct the environment structure.

Left image *I_l,t_* = [*u_l,p,t_ v_l,p,t_*]*^T^* is used to extract those pixels that may be interesting in the tracking process. Image edges from human contour, tables, doors, columns, and so on are visible and distinguishable from the background (even in quite crowded scenes) and can be easily extracted from the filtered image.

In order to robustly find structural features, the Canny image is zeroed in the Regions Of Interest (ROIs) where an obstacle is expected to appear. Therefore, the classification step is run over a partial Canny image, $I_{\mathrm{canny},l,t}={\left[u_{i,l,t}\;v_{i,l,t}\right]}_{i=1:\mathrm{mcanny}}^{T}$, though the full image is recovered to develop the 3D localization.

#### 2.1.2. Classification: Structural and Non-Structural Features

Within the partial Canny image *I_canny,l,t_*, edges corresponding with environmental structures have the characteristic of forming long lines. Thus, the classification process starts seeking structural shapes in the resulting image, through these typical features. Hough transform is used to search these long line segments in the partial Canny image.

The function *cvHoughLines2* [24] from OpenCV [25] library is used to accomplish the probabilistic Hough transform. This version of the Hough transform made by OpenCV allows finding line segments instead of whole ones if the image contains few long linear segments. This is the case of present application when obstacles in front of the camera set may occlude the structural elements of the scene.

This probabilistic version of Hough transform has five parameters to be tuned:

- *rho* and *theta* are respectively the basic Hough transform distance and angle resolution parameters in pixels and radians.
- *threshold* is the basic limit to overpass by the Hough accumulator in order to consider that a line exists.
- *length* is needed in the probabilistic version of Hough transform, and is the minimum line length, in pixels, for the detector of segments. This parameter is very important in the related work as it allows taking into account a line made by very short segments, like those generated in scenes with many occlusions.
- *gap* is also needed in the probabilistic version of Hough transform. This is the maximum gap in pixels between segment lines to be treated as a single line segment. This parameter is significant here, because it allows generating valid lines with very separated segments, due to occluding obstacles.

Due to the diversity of conditions that may appear in the experimental conditions an analytical study cannot be performed and thus all parameters have been empirically set. As a result of the challenging situation of obstacles in present application, not all lines related to structural elements in the environment are classified as structural features. In any case, the algorithm detects well enough the structural features existing in the scene: walls, columns, ceiling, floor, windows and so on. In the same way, it can also generate an obstacles features’ class neat enough to be used in the tracking step.

At the end of this classification step, two images are, therefore, obtained using the described process:

- $I_{\mathrm{structure},l,t}={\left[u_{i,l,t}\;v_{i,l,t}\right]}_{i=1:\mathrm{mstructure}}^{T}$ with the environmental structures, formed by the long lines found at the partial Canny image.
- $I_{\mathrm{obstacles},l,t}={\left[u_{i,l,t}\;v_{i,l,t}\right]}_{i=1:\mathrm{mobstacles}}^{T}$ with the full Canny image zeroed at the environmental structures.

### 2.2. 3D Localization of Structural and Obstacles’ Features

Both images are the inputs to a 3D localization process to obtain the 3D coordinates of structural $Y_{\mathrm{structure},t}={\left[x_{i,t}\;y_{i,t}\;z_{i,t}\right]}_{i=1:\mathrm{mstructure}}^{T}$ and obstacles’ features $Y_{\mathrm{obstacles},t}={\left[x_{i,t}\;y_{i,t}\;z_{i,t}\right]}_{i=1:\mathrm{mobstacles}}^{T}$. This is done in two phases by a matching process based on the epipolar geometry of the vision system; these phases are: 3D localization and obstacles’ features filtering.

#### 2.2.1. Phase 1: 3D Localization

Features’ classes *Y_structure,t_* and *Y_obstacles,t_* are respectively obtained calculating the ZNCC value for each non-zero pixel at the corresponding modified left images, *I_structure,l,t_* and *I_obstacles,l,t_* and using the full right image *I_r,t_*. Those features whose ZNCC values reaches a threshold are validated and finally classified in the corresponding features’ classes, *Y_structure,t_* or *Y_obstacles,t_*.

#### 2.2.2. Phase 2: Obstacles’ Features Filterin

Due to occlusions and repetitive patterns, correspondences between points in left and right images are often not correct and some outliers appear. This effect mainly affects to obstacles’ features. In order to reject these outliers, a neighborhood filter is run in the XZ plane over all points classified in the obstacles’ class *Y_obstacles,t_*.

**Figure 5.**
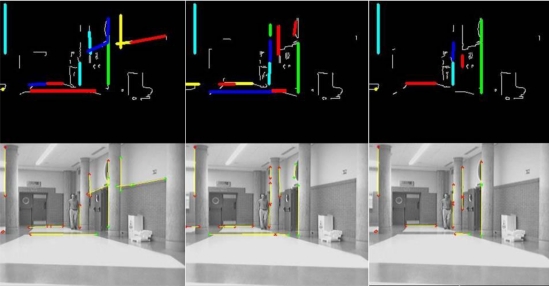
Results of the detection, classification and 3D location process in three frames of a real experiment. Detected structural features and related original images.

**Figure 6.**
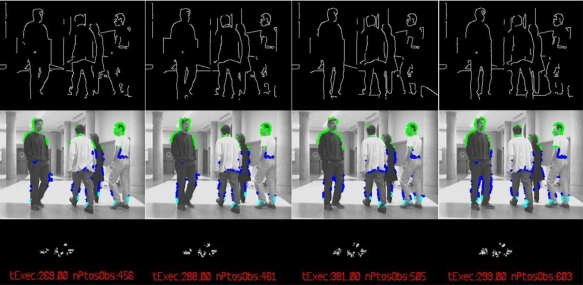
Results of the detection, classification and 3D location process in four frames of a real experiment. Top row, detected edges; middle row, original images; bottom row, 2D ground projection of points classified as obstacles.

The heights coordinate (Y) in each 3D position vector ${\left[x_{i,t}\;y_{i,t}\;z_{i,t}\right]}_{i=1:\mathrm{mobstacles}}^{T}$ is also used to filter the spurious noise. So, a feasible set of points that characterizes obstacles’ position in the scene is obtained in order to be used as measurement vector (observation model) at the posterior multiple obstacles’ tracking task (see Figure 2). Figure 5 and Figure 6 show some results obtained at the end of the whole detection, classification and 3D localization process.

Figure 5 shows a sequence of three frames belonging to a certain section of a single experiment. It is organized in two rows: the one at the top shows the results of the classification *I_structure,l,t_*. over the input Canny image *I_canny,l,t_* while the one at the bottom shows them over the original images. Those elements identified as members of the *structural features* class *Y_structure,t_* have been highlighted in both rows of images in order to show the behavior of the algorithm: in colors at the Canny image, and in yellow at the original image if their 3D localization ${\left[x_{i,t}\;y_{i,t}\;z_{i,t}\right]}_{i=1:\mathrm{mstructure}}^{T}$ has been found.

By the way, Figure 6 shows a different section of the same experiment. There are four frames in sequence from left to right organized in three rows. The row at the top shows the Canny image *I_canny,l,t_* input to the classification process; the central row shows the set of original images, where those 3D points ( ${\left[x_{i,t}\;y_{i,t}\;z_{i,t}\right]}_{i=1:\mathrm{mobstacles}}^{T}$) assigned to the *obstacles’ features* class *Y_obstacles,t_* are then projected back in colors according to their height in the Y coordinate (light blue for lower values, dark one for middle ones and green for higher ones). Finally, the row at the bottom is a 2D projection over the ground (XZ plane) of the set of points of the *obstacles’ features* class *Y_obstacles,t_*. The clouds of points in the 2D projection allow perform the tracking task of the four persons found in the original sequence.

In this last figure, it can be noticed that obstacles’ features *Y_obstacles,t_* related to the legs of the persons in the scene do not include all edge points related to them in the preliminary Canny image *I_canny,l,t_* Nevertheless, the multi-obstacles’ tracker works perfectly in any situation as it is demonstrated in the video *MTracker.avi* (see supplementary materials) from the experiment shown in Figure 6. In all the frames there are enough edge points in all obstacles, from 115 to 150 features per person to be tracked; the total amount of them are displayed at the bottom of each column in Figure 6 (parameter nPtosObs, text in red).

The difference between the points found in the Canny image and the final obstacles’ features class is related to the probabilistic Hough transformed used. As described in a previous section, the Hough algorithm is tuned to detect short segments of lines and classify them as structural features, in order to find them even in situations of high level of occlusion such the one displayed in Figure 6. Then, some linear features belonging to people arms or legs are sorted out to the structural class.

## 3. The Multiple Obstacles’ Tracker

As discussed in the introduction, a probabilistic algorithm is the best solution in order to implement the multi-obstacles tracking task. The XPFCP (eXtended Particle Filter with Clustering Process) an extended version of the PF has been chosen to develop this process in order to exploit its multimodality.

The combination of both techniques (probabilistic estimation and deterministic association) increases the robustness of the PF multimodality, a behavior which is difficult to develop when this combination is not used, as seen in [18]. In fact, the idea of combining probabilistic and deterministic techniques for tracking multiple objects has been proposed in different previous works, such as [6] or [26]. However none of them faced the idea of reinforcing the PF multimodality within the deterministic framework.

**Figure 7.**
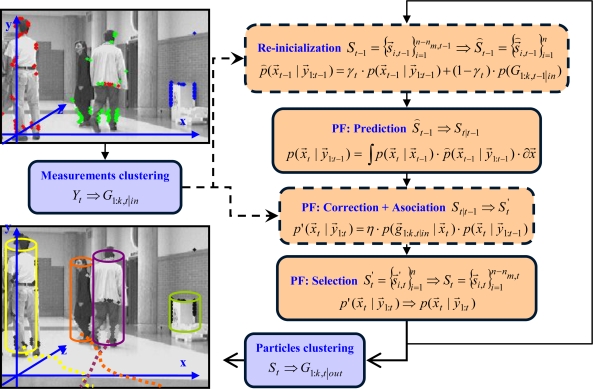
Functional diagram of the multiple objects’ tracker based on a XPFCP. Deterministic tasks have a blue background while probabilistic tasks have a different color. Modified or new PF steps are remarked with dashed lines.

Figure 7 shows a functional description of the multiple obstacles’ tracking algorithm proposed. As it can be noticed in the upper left corner of the figure, the input of the XPFCP is the obstacles’ features class *Y_obstacles,t_*: the set of measurements, unequally distributed among all obstacles in the scene, are clustered in a set of *k_in,t_* groups *G*_1:_*_k,t|in_* to work as observation density *p*(*y⃗_t_*) ≈ *p*(*G*_1:_*_k,t|in_*).

On the other hand, the image at the lower left corner in Figure 7 shows the output of the XPFCP based multi-obstacles tracking: a set of *k_out,t_* objects *G*_1:_*_k,t|out_* identified by colors with their corresponding location, speed and trajectory followed in the XYZ space.

The three standard steps of Bootstrap PF (prediction, correction and association) can also be seen in Figure 7. As shown in the figure, the PF implements a discrete representation of the belief *p*(*x⃗_t_* | *y⃗*_1:*t*_) with a set of *n* weighted samples $p({\vec{x}}_{t}|{\vec{y}}_{1:t})≅S_{t}={\{{\vec{s}}_{i,t}\}}_{i=1}^{n}=\{{{\vec{x}}_{t}^{\left(i\right)},w_{t}^{\left(i\right)}\}}_{i=1}^{n}$ (generally called particles) to develop the estimation task. Thanks to this kind of representation, different modes can be implemented in the discrete belief generated by the PF, which applied to the case of interest allow to characterize different tracked objects.

Besides, a new re-initialization step prior to the prediction one has also been included in the loop (dashed lines in Figure 7) in order to ease the generation of new modes in the *t* − 1 modified belief *p̑*(*x⃗*_*t*−1_|*y⃗*_1:*t*−1_) output by this step. As shown in this figure, this new re-initialization step is executed using the clusters segmented from the XPFCP input data set of obstacles’ features *G*_1:*k,t*−1|*in*_, therefore including in the tracking task a deterministic framework (blocks in blue in Figure 7).

The set *G*_1:*k,t|in*_, is also used at the correction step of the XPFCP, modifying the standard step of the Bootstrap PF, as displayed in Figure 7 (dashed lines). At this point, the clustering process works as a NN association one, reinforcing the preservation of multiple modes (as many as obstacles being tracked at each moment) in the output of the selection step: the final belief *p*(*x⃗_t_*|*y⃗*_1:*t*_).

The deterministic output *G*_1:*k,t|out*_ is obtained organizing in clusters the set of particles $S_{t}={\left\{{\vec{s}}_{i,t}\right\}}_{i=1}^{{n-n}_{m,t}}$ that characterizes the belief *p*(*x⃗_t_*|*y⃗*_1:*t*_) at the end of the XPFCP selection step. This new clustering process discriminates the different modes or maximum probability peaks in *p*(*x⃗_t_*|*y⃗*_1:*t*_), representing the state *x⃗_t_* of all *k_out,t_* objects being tracked by the probabilistic filter at that moment. The following subsections extend the description of XPFCP functionality.

### 3.1. The Tracking Model

The application of the XPFCP to the position estimation problem requires a model definition. In the application of interest, a Constant Velocity (CV) model is used [27], where the actuation and observation models are defined by equation (1) and equation (2), respectively:

(1) $${\vec{x}}_{t|t-1}=\left[\begin{array}{c} x_{t|t-1}\\ y_{t|t-1}\\ z_{t|t-1}\\ {\dot{x}}_{t|t-1}\\ {\dot{z}}_{t|t-1} \end{array}\right]=\left[\begin{array}{ccccc} 1 & 0 & 0 & t_{s} & 0\\ 0 & 1 & 0 & 0 & 0\\ 0 & 0 & 1 & 0 & t_{s}\\ 0 & 0 & 0 & 1 & 0\\ 0 & 0 & 0 & 0 & 1 \end{array}\right]\cdot \left[\begin{array}{c} x_{t-1}\\ y_{t-1}\\ z_{t-1}\\ {\dot{x}}_{t-1}\\ {\dot{z}}_{t-1} \end{array}\right]+{\vec{v}}_{t-1}$$

(2) $${\vec{y}}_{t}=\left[\begin{array}{c} x_{t}\\ y_{t}\\ z_{t} \end{array}\right]=\left[\begin{array}{ccccc} 1 & 0 & 0 & 0 & 0\\ 0 & 1 & 0 & 0 & 0\\ 0 & 0 & 1 & 0 & 0 \end{array}\right]\cdot \left[\begin{array}{c} x_{t}\\ y_{t}\\ z_{t}\\ vx_{t}\\ vz_{t} \end{array}\right]+{\vec{o}}_{t}$$

As shown in equation (1), the estimation vector *x⃗*_*t|t−*1_ will define the position and speed state of the obstacle being tracked. In addition, the state noise vector *v⃗_t_* (empirically characterized as Gaussian and white) is included in the actuation model both to modify the constant speed of the obstacle, and to model the uncertainty related to the probabilistic estimation process.

Furthermore in equation (2), *y⃗_t_* defines the observable part of the state *x⃗*_*t|t−*1_, that in this case matches with the 3D position information ( $Y_{\mathrm{obstacles},t}={\left[x_{i,t}\;y_{i,t}\;z_{i,t}\right]}_{i=1:\mathrm{mobstacles}}^{T}$) extracted by the stereo vision process described in section 2. An observation noise vector *o⃗_t_* has also been included to model the noise related to that vision process, and so, it is characterized in an off-line previous step. This noise model makes possible to keep tracking objects when they are partially occluded.

Empirical studies over tests results, including different environmental and tracking conditions, were used to identify the standard deviation of all components in *v⃗_t_* and in *o⃗_t_*, resulting that σ*_v,i_* = 100*mm* / *i* = {*x, y, z, ẋ, ż*} and σ*_o,i_* = [150,200]*mm*/*i* = {*x,y,z*}. Besides, the study of sensibility concluded that a modification of a 100% in any of σ*_o,i_* generates an increase in the tracking error of around 24%, while the same modification in any of σ*_v,i_* generates ten times lower figures. This result indicates the importance of the observation noise vector in the multi-obstacles’ tracking task.

### 3.2. Steps of the XPFCP

#### 3.2.1. Clustering Measurements

The clustering process is done over the 3D position data set *Y_obstacles,t_* extracted by the stereo vision process. The output set of groups *G*_1:*k,t|in*_ generated by this process is then used in the re-initialization and correction steps of the XPFCP.

**Figure 8.**
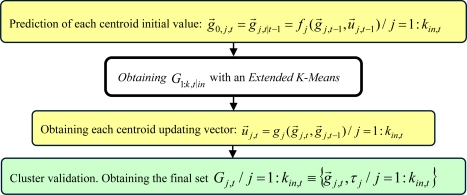
Functional diagram of the modified version of the Extended K-Means (second step, white background), used in the correction step of the XPFCP: the *Sequential K-Means with Validation*. New steps of this clustering algorithm are highlighted in yellow and green.

We propose an adapted version of Extended K-Means [28] to solve this clustering task, called *Sequential K-Means with Validation*; a general description of it is presented in Figure 8. The simplicity and reliability of this clustering process ensures a correct re-initialization and association tasks in the XPFCP, within a low computational load that makes possible a real time execution of the global tracking task, as reveal the results obtained in our tests.

The main characteristics of this clustering proposal are listed below; while a deeper description of it can be found in [28]:

- The clustering algorithm adapts itself to an unknown and variable number *k_in,t_* clusters, as needed in this application.
- A preliminary centroid *g⃗*_1:*k,t|in*_ prediction is included in the process in order to make fast and sure its convergence (the execution time of the proposal is decreased in 75% related to the standard K-Means’s one). This centroid prediction is possible thanks to the first and third steps of the blocks diagram in Figure 8: predicting an initial value for each centroid *g⃗*_0,1:*k,t|in*_, and computing each centroid updating vector *u⃗*_1:*k,t|in*_.
- A window based validation process is added to the clustering proposal in order to increase its robustness against outliers in almost a noise rejection rate of 70%. Besides, this process provides the inclusion of an identifier τ_1:*k|out*_ for each cluster obtained, with a 99% success rate meanwhile the cluster keeps appearing among the input data set *Y_obstacles,t_*. Thanks to this functionality, the validation process (last step, remarked in green in Figure 8) helps keeping track of temporal total occlusions of objects in the scene, as it is demonstrated in the video sequence *MTracker.avi* (see supplementary materials).

With these characteristics the set *G*_1:*k,t|in*_ ≡ {*g⃗_j,t_*, τ*_j_* / *j* = 1 : *k_in,t_*} comprises a robust, filtered, compact and identified representation of the corresponding input data, which strengths the PF reliability in the multimodal estimation task pursuit.

#### 3.2.2. Re-Initialization

The main aim of adding the re-initialization step to the standard Bootstrap PF, is to insert *n*_*m,t*−1_ new particles to the discrete belief *S*_*t*−1_ ≅ *p*(*x⃗*_*t*−1_|*y⃗*_1:*t*−1_) from time *t* − 1. So, new tracking events (inclusion or loss of any object in the scene) are quickly updated in the estimation process.

Particles inserted in this new step are obtained randomly sampling among the members of all *k*_*in,t*−1_ clusters G_1:*k,t*−1|*in*_, segmented from the input data set of obstacles’ features *Y*_*obstacles,t*−1_. Therefore, the re-initialization step generates the discrete density *S̑*_*t*−1_ ≅ *p̑*(*x⃗*_*t*−1_|*y⃗*_1:*t*−1_), which is a modification of *S*_*t*−1_ ≅ *p*(*x⃗*_*t*−1_|*y⃗*_1:*t*−1_) described by equation (3):

(3) $${\overset{⌢}{S}}_{t-1}=\overset{k_{\mathrm{in},t-1}}{\underset{j=1}{\cup }}S_{t-1},\;f(G_{j,t-1|\mathrm{in}})$$

This process ensures that all observation hypotheses modeled by the density *p*(*G*_1:*k,t*−1|*in*_) are considered equally in the re-initialization process.

In order to increase the probability of newly sensed objects in *S̑*_*t*−1_, a specific number of particles *n*_*m*|*i t*−1_ is defined for each cluster *j* = 1: *k*_*in,t*−1_ to be inserted at this step, as shown in equation (4):

(4) $$n_{m,t-1}=\sum_{j=1}^{k_{\mathrm{in},t-1}}n_{m|j,t-1}=\sum_{j=1}^{k_{in,t-1}}\left(n_{m}+n_{\mathrm{init}}\cdot \alpha_{\mathrm{init},j,t-1}\right)$$

where *α*_*init,j,t*−1_ is a boolean parameter informing about the novelty of the cluster *G*_*j,t*−1|*in*_ in the set *G*_1:*k,t*−1|*in*_; *n_init_* is the number of particles to append for each new cluster; *n_m_* is the minimum number of particles per cluster to be included; and *n*_*m,t*−1_ is the total amount of particles inserted at this step in *S*_*t*−1_ to get *S̑*_*t*−1_.

Besides, γt=nm,t−1n relates the number of particles inserted at re-initialization step *n*_*m,t*−1_ with the number *n* of them obtained at the output of this step. Using *γ_t_* a continuous version of equation (3) can be expressed as shown in equation (4) and in Figure 7:

(5) $$\overset{⌢}{p}({\vec{x}}_{t-1}|{\vec{y}}_{1:t-1})=\gamma_{t}\cdot p({\vec{x}}_{t-1}|{\bar{y}}_{1:t-1})+(1-\gamma_{t})\cdot p(G_{1:k,t-1|in})$$

The deterministic specification of *n*_*m|j,t*−1_ for each *j* = 1:*k*_*in,t*−1_ helps shortcoming the impoverishment problem of the PF in its multimodal application. This process ensures the particles diversification among all tracking hypotheses in the density estimated by the PF and increases the probability of newest ones, that otherwise would disappear along the filter evolution. Results included in section 4 demonstrates this assertion for a quite low value of γ*_t_*, that maintains the mathematical recursive rigor of the Bayesian algorithm.

This re-initialization step has a similar behavior that the one of the MCMC step (used *i.e.*, in [15]) which moves the discrete density *p̑*(*x⃗*_*t*−1_|*y⃗*_1:*t*__−1_) towards high likelihood areas in the probability space. In order to maintain constant the number of particles in *S_t_* along the time (and thus the XPFCP execution time), the *n*_*m,t*−1_ of them that are to be inserted at the re-initialization step at time are wisely erased at the selection step at time *t* − 1.

#### 3.2.3. Prediction

The set of *n* particles generated by the re-initialization step *S̑*_*t*−1_ ≅ *p̑*(*x⃗*_*t*−1_|*y⃗*_1:*t*__−1_) is updated through the actuation model, to obtain a discrete version of the prior *S*_*t|t*−1_ ≅ *p*(*x⃗_t_*|*y⃗*_1:*t*−1_).

(6) p(x→t|y→1:t−1)=∫p(x→t|x→t−1)⋅p⌢(x→t−1|y→1:t−1)⋅∂x→≅St|t−1St|t−1={x→t|t−1(i),1n}i=1n→x→t|t−1(i)=p(x→t|x→t−1)⋅x→⌢t−1(i)

In this case, the actuation model used *p*(*x⃗_t_*|*x⃗*_*t*−1_) is defined in section 3.1, and so, the last expression in equation (6) can be replaced by equation (1).

Thus, the state noise component *v⃗*_*t*−1_ is included in the particles’ state prediction with two main objectives: to create a small dispersion of the particles in the state space (needed to avoid degeneracy problems of the set [9]); and a slight modification of the speed components in the state vector (needed to provide movement to the tracking hypothesis when using the CV model [27]).

The simplicity of the CV model proposed eases its use for all objects to be tracked, no care its type or dynamics and without the help of an association task. Each particle ${\vec{s}}_{i,t}={\left\{{\vec{x}}_{t}^{\left(i\right)},w_{t}^{\left(i\right)}\right\}}_{i=1}^{n}/i=1:n$ evolves according to the object’s dynamics that represents in the belief, as the related state vector includes the object speed components.

#### 3.2.4. Correction and Association

Particles’ weights ${\vec{w}}_{t}={\left[{\tilde{w}}_{t}^{\left(i\right)}\right]}_{i=1}^{n}$ are computed at the correction step, using the expressions at equation (7), including a final normalization:

(7) wt(i)=wt−1(i)⋅p(g→1:k,t|in|x→t(i)=wt−1(i)⋅e−dmin⁡,i,t22⋅O/i=1:nw˜t(i)=wt(i)∑i=1nwt(i)/i=1:ndmin⁡,i,t=min⁡j=1:k{d(h(x→t|t−1(i)),g→j,t|in)}/i=1:n

where *d*_min,*i,t*_ is the shortest distance in the observation space (XYZ in this case), for particle *S⃗*_*i,t|t*−1_, between the projection in this space of the predicted state vector represented by the particle $h({\vec{x}}_{t|t-1}^{\left(i\right)})$, and all centroids *g⃗*_1:*k,t|in*_ in the cluster set *G*_1:*k,t|in*_, obtained from the objects’ observations set *Y_obstacles,t_*. The use of cluster centroids guarantees that the observation model applied is filtered, robust and accurate whatever the reliability of the observed object.

As shown in equation (7), in order to obtain the likelihood $p({\vec{g}}_{1:k,t|in}|{\vec{x}}_{t}^{\left(i\right)})$ used to compute the weights array *w⃗_t_*, the observation model defined by (2) has to be utilized, as $h\left({\vec{x}}_{t|t-1}^{\left(i\right)}\right)={\vec{y}}_{t}^{\left(i\right)}$. Besides, *O* is the covariance matrix that characterizes the observation noise defined in the same model. This noise models the modifications of positions in the clusters *G_j,t|in_* centroid *g⃗*_*j,t|in*_, when tracking objects that are partially occluded.

The equally weighted set St|t−1={x→t|t−1(i),1n}i=1n output from the prediction step is therefore converted in the set $S_{t}^{\prime }={\left\{{\vec{x}}_{t|t-1}^{\left(i\right)},{\tilde{w}}_{t}^{\left(i\right)}\right\}}_{i=1}^{n}$.

The mentioned definition of *d*_min,*i,t*_ involves a NN association between the cluster, *G_j,t|in_*, whose centroid *g⃗_j,t|in_* is used in the particle’s weight ${\tilde{w}}_{t}^{\left(i\right)}$ computation and the tracking hypothesis represented by the particle *S⃗*_*i,t|t*−1_ itself. In fact, this association means that *g⃗*_*j,t|in*_ is obtained from the observations generated by the tracking hypothesis represented by *S⃗*_*i,t|t*−1_.

This association procedure and the re-initialization step remove the impoverishment problem that appears when a single PF is used to estimate different state vector values: all particles tend to be concentrated next to the most probable one, leaving the rest of its values without probabilistic representation at the output density. In [17], the approximate number of efficient particles *n̑_eff_* is used as a quality factor to evaluate the efficiency of every particle in the set. According this factor, *n̑_eff_* should be above 66% in order to prevent the impoverishment risk at the particle set. This parameter is included among the results presented in next section in order to demonstrate how the XPFCP solves the impoverishment problem.

#### 3.2.5. Selection

Each particle of the set $S_{t}^{\prime }={\left\{{\vec{x}}_{t|t-1}^{\left(i\right)},{\tilde{w}}_{t}^{\left(i\right)}\right\}}_{i=1}^{n}≅p^{\prime }\left(x_{t}|y_{1:t}\right)$ output from the correction step is resampled at the selection step (also called resampling step) according to the generated weight. As a result, an equally weighted particle set St={x→t(i),1(n−nm,t)}i=1n−nm,t is obtained, representing a discrete version of the final belief estimated by the Bayes filter *p*(*x_t_*|*y*_1:*t*_). This final set *S_t_* is formed by *n* − *n_m,t_* particles, in order to have *n_m,t_* inserted at the next re-initialization step.

#### 3.2.6. Clustering Particles

From the discrete probabilistic distribution *S_t_* ≅ *p*(*x_t_*|*y*_1:*t*_) output by the selection step, a deterministic solution has to be generated by the XPFCP. This problem consists on finding the different modes included in the multimodal density *p*(*x_t_*|*y*_1:*t*_) represented by the particle set *S_t_*; it has not an easy solution if those modes are not clearly different in that distribution.

Diverse proposals have been included in the XPFCP in order to achieve this differentiation. This is because keeping this multimodality in *p*(*x_t_*|*y*_1:*t*_), while avoiding impoverishment problems in it, is the principal aim of all techniques proposed in this paper. Following section shows empirical results that demonstrates this.

Once ensured the differentiation, a simple algorithm can be used to segment in clusters the belief *p*(*x_t_*|*y*_1:*t*_) at the end of the XPFCP loop. Therefore, these groups *G*_1:*k,t|out*_ will become the deterministic representation of the multiple obstacles’ hypotheses *Y_obstacles,t_* detected by the stereo vision algorithm described in Section 2.

In this work, the same *Sequential K-Means with Validation*, described in Figure 8, is used in order to obtain *G*_1:*k,t|out*_ from *S_t_*. Therefore, the deterministic representation of each *j* = 1 : *k_out,t_* tracked hypothesis will be a cluster *G_j,t|out_* with centroid *g⃗_j,t|out_*, with the same components as the state vector defined in (1), and an identification parameter *τ_j|out_*.

## 4. Results

Different tests have been done in unstructured indoor environments, whose results are shown in this section. The stereo vision system used in the experiments is formed by two black and white digital cameras located in a static mounting arrangement, with a gap of 30 cm between them, and at a height of around 1.5 m from the floor. Vision processes have been developed using OpenCV libraries [25] and run on a general purpose computer (Intel DUO 1.8GHz).

The global tracking algorithm described in this paper has been implemented on a mobile 4-wheeled robot platform. Specifically a Pioneer2AT from MobileRobots© [29] has been used for the different tests. The robot includes a control interface to be guided around the environment, which can be used within the Player Control GNU Software, from the Player Project [30].

**Figure 9.**
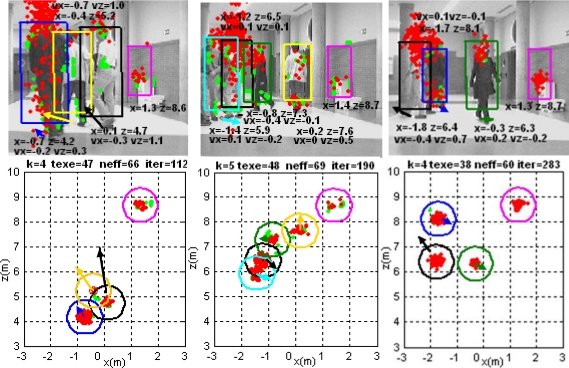
Results of the multi-tracking process in a real experiment. They are organized in columns, where the upper image shows the tracking results generated by the XPFCP for each object, projected in the image plane, and the lower one shows the same results projected into the XZ plane.

Figure 9 displays the functionality of the multi-tracking process in one of the tested situations. Three instants of the same experiment are shown in the figure. Each column presents the results obtained from a single capture; upper row are the input images, while lower row are 2D representations of objects’ data over the XZ ground plane.

Different data coming from the detected objects are found into each plot. According to the identification generated by the output clustering process, each group *G*_1:*k,t|out*_ has got a different and unique color. These groups are identified with a cylinder, thus this is shown as rectangles in the images and as circles in the ground projections. In both graphics, an arrow (with the same color than the corresponding group) shows the estimated speed of every obstacle being tracked at each situation, both in magnitude and in direction.

Particles’ state ${\vec{x}}_{t}^{\left(n-n_{m,t}\right)}$ (taken from the final set *S_t_* generated by the XPFCP) and 3D position of data set *Y_obstacles,t_* are represented by red and green dots, respectively, in each plot. Besides, the estimated values of position and speed (if non zero) of each obstacle are also depicted below its appearance in top row images.

Between any two plots in each column, a text row displays some information about the results shown; this is: the number of tracked obstacles (k); the execution time of the whole tracking application in ms (texe), the percent of *n̑_eff_* (neff) and the frame number in the video sequence (iter). As it can be noticed in Figure 9, the observation system proposed and described in section 2 performs correctly its detection, classification and 3D localization task. Every object not belonging to the environmental structure is detected, localized and classified in the obstacle data set *Y_obstacles,t_*, in order to be tracked afterwards.

The multimodal algorithm also achieves the position estimation objective for all obstacles in the scene, regardless the number, shape, dynamics and type of the object. The XPFCP correctly tracks deformable and dynamic objects, such us persons, and static ones such us the paper bin, which can be seen besides the wall on the right.

Moreover, each tracked object characterized by the corresponding particles’ cluster *G*_1:*k,t|out*_ maintains its identity *τ*_1:*k|out*_ (shown with the same color in Figure 9) while the object stays in the scene even if it is partially or totally occluded (for a certain time) to the vision system. This is possible thanks to the particles’ clustering algorithm that includes a window based validation process.

**Figure 10.**
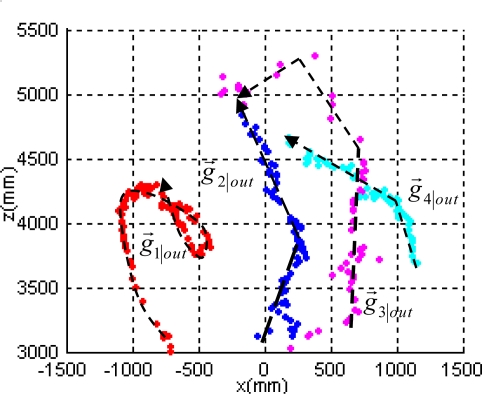
Trajectory followed in the ground plane (XZ) by four obstacles according to the XPFCP estimation results in a real experiment.

In order to show in detail the behavior of the identification task, Figure 10 shows the trajectories followed in the XZ plane by the four obstacles detected in another experiment. The robot stays stopped in front of the obstacles for the whole test.

Each colored spot represents during consecutive iterations the centroid position *g⃗*_1:4|*out*_ of the cluster related to the corresponding obstacle *G*_1:4*,t|out*_; each color reflects the cluster identity *τ*_1:4|*out*_. A dashed oriented arrow over each *g⃗*_1:4|*out*_ trace illustrates the ground truth of the path followed by the real obstacles. It can be hence conclude, that the correct identification of each object *τ*_1:4|*out*_ is maintained with a 100% of reliability, even when partial and total occlusions occur; this is the case shown on traces from obstacles three (in pink) and four (in light blue).

**Figure 11.**
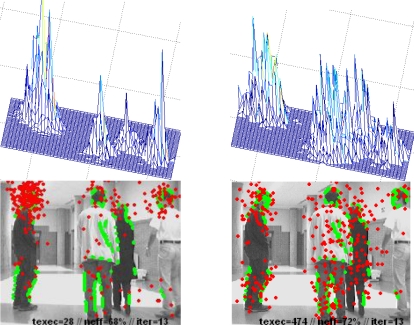
Results of the multi-tracking process in a real experiment: left column shows the results generated by the XPFCP; the right column shows the results of the proposal presented in [18].

Figure 11 demonstrates graphically the multimodal capability of the XPFCP proposal in a multi-tracking task. In this figure, the XPFCP functionality is compared to that of another multimodal multi-tracking proposal, described in [18].

The bottom row of images in Figure 11 shows the same particles and observation data set projections, as well as the tracking parameters texe, neff and iter, as described for Figure 9. Besides, the top row includes a plot of the density represented by the set output from the correction step by the two algorithms.

The information included in Figure 11 allows concluding that the XPFCP proposed (left column) generates well differentiated modes in the final belief, according to the different estimation hypotheses; this is shown with four clear peaks on the belief distribution (top row). However, the PF based multi-tracking proposal presented in [18] does not achieve the multimodality objective with the same efficiency than XPFCP, and therefore it cannot be used to robustly track multiple objects within a single estimator.

As theoretically asserted in previous sections, the measurements clustering algorithm used as deterministic association process have better results in the multimodal estimation task. Moreover, the results presented in Figure 11 show that the multimodal density obtained with the XPFCP *S_t_* ≅ *p*(*x⃗_t_*|*y⃗*_1:*t*_), can be easily segmented to generate a deterministic output *G*_1:*k,t|t*_, which is not the case with the results generated by the proposal in [18]. A fast clustering algorithm, like the K-Means based proposed in this work, is enough to fulfill this task robustly and with low execution time. As it can be seen in the figure, the execution time of the XPFCP (texe = 28 ms) is almost 17 times smaller than the one of the other algorithm (texe = 474 ms); therefore, the Bayesian proposal presented in this paper is more appropriate for a real time application than the proposal in [18].

**Figure 12.**
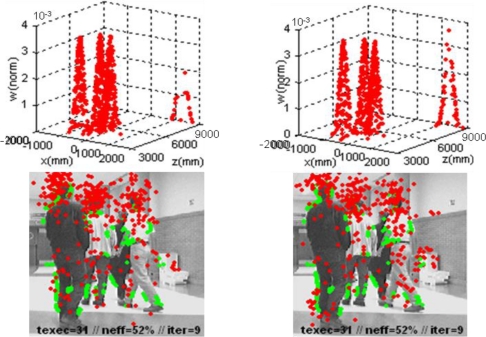
Results of the multi-tracking process in a real experiment using the proposed XPFCP (left column of images), and the same results using an input data set not segmented in classes at the re-initialization and correction steps (right column of images).

Finally, the data shown in Figure 12 confirm that the impoverishment problem related to the Bootstrap filter is minimized using the observation data set *Y_obstacles,t_* organized in clusters *G*_1:*k,t|in*_ at the re-initialization and correction steps. The bottom row of images in Figure 12 shows the same information and parameters than the corresponding one in Figure 11. By the other side, the upper row plots the weights array ${\vec{w}}_{t}={\left[{\tilde{w}}_{t}^{\left(i\right)}\right]}_{i=1}^{n}$ output from the correction step. Analyzing the results included in Figure 12, it is concluded that if the proposed segmentation in *G*_1:*k,t|in*_ clases is not used (left column plots) the poorest sensed object in the scene (the paper bin besides the wall on the right), has a reduced representation in the discrete distribution output of the correction step $S_{t}^{\prime }={\left\{{\vec{x}}_{t|t-1}^{\left(i\right)},{\tilde{w}}_{t}^{\left(i\right)}\right\}}_{i=1}^{n}$. However, results generated by the XPFCP in the same situation (right column plots) are much better. A visual comparison between both discrete distribution plots (top row) show the claimed behavior.

In order to analyze quantitatively this situation, Table 1 shows the number of particles in the set (output from the selection step) assigned to each object in the scene in Figure 12, numbered according its position in the image from left to right.

From the figures shown in Table 1, It can be seen that particles are more equally distributed among all tracking hypotheses when using at the re-initialization and correction steps, avoiding the mentioned impoverishment problem.

As a final analysis, Table 2 resumes the results obtained with the proposed system (XPFCP with stereo vision data input) in a long experiment of 1,098 frames (video sequence of 1 min 13 s) with complex situations similar to the ones presented in Figure 9. The number of obstacles in the scene is changing from 0 to 5 along the sequence.

Table 2 data allow concluding that the multi-tracking proposal achieves the proposed objective reliably and robustly:

- The low computational load of the tracking application enables its real time execution.
- The impoverishment problem has been correctly solved because the number of efficient particles involved in the PF is above the established threshold (66%).
- The XPFCP shows high identification reliability and robustness against noise.
- A detailed analysis of tracking reliability shows errors (missed, duplicated or displaced objects) in about a 13% of iterations.
- Nevertheless, noticeable errors in the tracking application (those of more than three consecutive iterations) only reached a 5.3% of iterations in the whole experiment.

## 5. Conclusions

A robust estimator of the movement of obstacles in unstructured and indoor environments has been designed and tested. The proposed XPFCP is based on a probabilistic multimodal filter and is completed with a clustering process. The algorithm presented in this paper, provides high accuracy and robustness in the tracking task in complex environments, and obtains better figures than other up-to-date proposals.

As well, it has been developed a specific detection, classification and 3D localization algorithm for a stereo vision observation system. This algorithm is able to handle those tasks in a dynamic and complex indoor environment. The designed algorithm makes also a separation in real time of the measurements acquired from obstacles from those acquired from structural elements belonging to the environment.

The input data to the detection and classification process are stereo vision images, coming from a pair of synchronized cameras. The vision system has demonstrated to be robust in different scenes and distances up to 20 m.

Results obtained with the proposed algorithm are shown throughout this article. They prove that the exposed objectives have been achieved robustly and efficiently. The reliability shown by these results is especially important as the system is thought to be used in tracking applications for autonomous robot navigation.

To track a variable number of objects within a single algorithm, an estimator called XPFCP has been specified, developed and tested. In order to achieve this multimodal behavior, a combination of probabilistic and deterministic techniques has been successfully used.

The XPFCP includes a deterministic clustering process in order to increase the likelihood hypothesis of new objects appearing on the scene. This clustering improves the robustness of XPFCP compared with the behavior shown by other multimodal estimators.

Most tests have been run with a fixed number of 600 particles. This figure is kept constant so the XPFCP execution time is also constant; this is a very important fact in order to achieve a real time performance.

The designed XPFCP is based on simple observation and actuation models, and therefore it can be easily adapted to handle data coming up from different kinds of sensors and different types of obstacles to be tracked. This fact demonstrates that our tracking proposal is more flexible than other solutions found in the related literature, based on rigid models for the input data set.

## Supplemental Information



## Figures and Tables

**Table 1.** Distribution percentage of particles in the set *S_t_* among the tracked hypotheses in the situations shown in Figure 12.

**Algorithm**	**Object**			
	**1**	**2**	**3**	**4**
Using *G*_1:*k,t*−1|*in*_ (left column plots)	28.5	28.1	31.5	10.9
Not using *G*_1:*k,t*−1|*in*_ (right column plots)	31.2	42.2	24.4	2.2

**Table 2.** Summary of the results obtained with the multi-tracking proposal in a long and complex experiment. The most relevant parameters in the XPFCP are tuned to the values: *n* = 600, *γ_t_* = 0.2, ninitn=5%, σ*_v,i_* = 100 / *i* = {*x,y,z,vx,vz*}, σ*_o,i_* = 150*mm* / *i* = {*x,y,z*}.

**Parameter**	**Value**

Mean execution time	40 ms (25 FPS)
Number of efficient particles, *n̑_eff_*	69.8%
Mismatch identification (% frames)	0%
Outliers rejection (% frames)	99.9%
Missed objects (% frames)	9.2%
Duplicated objects (% frames)	3.3%
Displaced objects (% frames)	0.4%
Reliability in long term errors (% frames)	Δ*t* > 0.6s → 3.5%, Δ*t* > 0.8s → 1.8%
